# Large Genomic Rearrangements of *BRCA1* and *BRCA2* among Patients Referred for Genetic Analysis in Galicia (NW Spain): Delimitation and Mechanism of Three Novel *BRCA1* Rearrangements

**DOI:** 10.1371/journal.pone.0093306

**Published:** 2014-03-31

**Authors:** Laura Fachal, Ana Blanco, Marta Santamariña, Angel Carracedo, Ana Vega

**Affiliations:** 1 Fundación Pública Galega de Medicina Xenómica-SERGAS. Grupo de Medicina Xenómica, CIBERER, IDIS, Santiago de Compostela, Spain; 2 Grupo de Medicina Xenómica -USC, University of Santiago de Compostela, CIBERER, IDIS, Santiago de Compostela, Spain; Ohio State University Medical Center, United States of America

## Abstract

In the Iberian Peninsula, which includes mainly Spain and Portugal, large genomic rearrangements (LGRs) of *BRCA1* and *BRCA2* have respectively been found in up to 2.33% and 8.4% of families with hereditary breast and/or ovarian cancer (HBOC) that lack point mutations and small indels. In Galicia (Northwest Spain), the spectrum and frequency of *BRCA1/BRCA2* point mutations differs from the rest of the Iberian populations. However, to date there are no Galician frequency reports of *BRCA1/BRCA2* LGRs. Here we used multiplex ligation-dependent probe amplification (MLPA) to screen 651 Galician index cases (out of the 830 individuals referred for genetic analysis) without point mutations or small indels. We identified three different *BRCA1* LGRs in four families. Two of them have been previously classified as pathogenic LGRs: the complete deletion of *BRCA1* (identified in two unrelated families) and the deletion of exons 1 to 13. We also identified the duplication of exons 1 and 2 that is a LGR with unknown pathogenicity. Determination of the breakpoints of the *BRCA1* LGRs using CNV/SNP arrays and sequencing identified them as NG_005905.2:g.70536_180359del, NG_005905.2:g.90012_97270dup, and NC_000017.10:g.41230935_41399840delinsAluSx1, respectively; previous observations of *BRCA1* exon1-24del, exon1-2dup, and exon1-13del LGRs have not characterized them in such detail. All the *BRCA1* LGRs arose from unequal homologous recombination events involving Alu elements. We also detected, by sequencing, one *BRCA2* LGR, the Portuguese founder mutation c.156_157insAluYa5. The low frequency of *BRCA1* LGRs within *BRCA1* mutation carriers in Galicia (2.34%, 95% CI: 0.61–7.22) seems to differ from the Spanish population (9.93%, 95% CI: 6.76–14.27, *P*-value = 0.013) and from the rest of the Iberian population (9.76%, 95% CI: 6.69–13.94, *P*-value = 0.014).

## Introduction

The two major high-penetrance breast cancer susceptibility genes, *BRCA1* [1] and *BRCA2* [2], account for approximately 26% of all cases of hereditary breast and/or ovarian cancer (HBOC) [3]. To date, some 1,700 *BRCA1* variants and 1,900 *BRCA2* variants have been reported (Breast Cancer Information Core database, http://research.nhgri.nih.gov/bic/). However, only 81 *BRCA1* variants and 17 *BRCA2* variants are large genomic rearrangements (LGRs) [4], and the prevalence of *BRCA1/2* LGRs varies widely among different populations, mainly due to the existence of founder rearrangements. For example, in the Netherlands, *BRCA1* LGRs constitute up to 27% of all *BRCA1* mutations (see Sluiter et al. [4] and references therein), whereas to date, only one *BRCA2* LGR has been reported in a proband of Dutch and German ancestry [5]. On the contrary, in Portugal *BRCA1* LGRs represents the ∼6% of *BRCA1* mutations while, due to the Portuguese *BRCA2* founder mutation c.156_157insAluYa5, the frequency of *BRCA2* LGRs is the highest reported to date (57.89% of *BRCA2* mutations) [6]–[8].

Of the published studies of the frequency of *BRCA1*/*2* LGRs in the Iberian Peninsula or regions thereof [6]–[17], none has specifically examined the population of Galicia (NW Spain), a region with a distinct genetic identity attributable to its historical relative isolation, its cultural identity, and the occurrence of a marked population bottleneck around 1000 years ago [18]. This population features a number of founder mutations [18]–[20], including a *BRCA1* mutation, c.211A>G (referred to NM_007294.3; BIC 330A>G), which is present in more than 50% of Galician HBOC families with *BRCA1/2* mutations [21].

In view of to date there are no Galician frequency reports of *BRCA1/BRCA2* LGRs and the observed differences between Galicia and the rest of Spain in regard to the spectrum and prevalence of point mutations of *BRCA1* and *BRCA2*, we decided to investigate whether a similar situation holds for *BRCA1/2* LGRs. We accordingly screened for *BRCA1/2* LGRs among Galician families referred for genetic examination of *BRCA1/2*. Here we describe three novel *BRCA1* LGRs, propose likely originating mechanisms, and compare the frequency of LGRs in Galicia with published results for the remainder of the Iberian Peninsula.

## Materials and Methods

### Participants

Our reference laboratory handles essentially all Galician patients referred for evaluation of the possibility of HBOC by means of DNA analysis. Between 1997 and 2012 we examined *BRCA1/2* in 830 patients referred to us in accordance with the criteria established in Galician oncological guidelines [22], which currently recommend referral if patients have (i) three or more first degree relatives who have suffered breast or ovarian cancer, (ii) two affected first degree relatives if one was aged <40 years at diagnosis, (iii) two affected first or second degree relatives if both suffered breast cancer at age <50 years, or if one suffered bilateral breast cancer and one was aged <50 years at diagnosis, or if at least one of these cancers was ovarian or a male breast cancer, (iv) age <30 years at diagnosis of a breast cancer, (v) both breast and ovarian cancer, (vi) bilateral breast cancer diagnosed before the age of 40 years, or (vii) a family history of deleterious mutation of a breast cancer susceptibility gene. These patients were first screened for the founder mutations *BRCA2* c.156_157insAluYa5 and *BRCA1* c.211A>G (BIC 330A>G) using protocols respectively described by Peixoto et al. [7] and Vega et al. [21], after which other *BRCA1/2* mutations were sought by bi-directional sequencing of exons and flanking intronic splice sites. Of the 830 referred patients, 125 were found to have point mutations or small indels in *BRCA1*, and 54 point mutations or small indels in *BRCA2*. The 651 with no point mutations or small indels were included in the present study, as were members of their families when this was appropriate and possible.

Relationships between index cases were investigated through the genealogical tree, which include at least three generations, and the family name that in Spain included the surname from the father and also from the mother.

### Ethics Statement

The study conformed to Spanish biomedical research legislation (*Ley* 14/2007) and was approved by the Galician Ethical Committee for Clinical Research. All participants gave written informed consent.

### LGR screening

Screening for LGRs in *BRCA1* and *BRCA2* was performed by multiplex ligation-dependent probe amplification (MLPA). The commercial *BRCA1* kits P002 (primary screening) and P087 (confirmatory) and the *BRCA2* kit P045 were used in accordance with the manufacturer's instructions [23]. Fragment electrophoresis was performed on an Applied Biosystems 3730 xl DNA analyzer using GeneScan 500 LIZ size standards (Applied Biosystems, USA) and at least 24 samples in each run, and the resulting data were analyzed using GeneMapper software (Applied Biosystems, USA). Visual peak pattern evaluation was carried out following the manufacturer's recommendations. After exclusion of samples failing the first quality control, the remaining samples were analyzed using Coffalyser v8 software in “direct analysis” normalization mode using concurrently run samples as the reference set and taking the medians of the corresponding normalized probe signal ratios. When all sample signals had been normalized in this way, any samples with aberrant probe signals (>0.15 standard deviations from the mean) were removed, the whole normalization process was repeated, and so on until the standard deviations of all probe signals were <0.15.

### SNP arrays and analysis of breakpoint regions

LGRs were characterized using the Cytogenetics Whole-Genome 2.7 M Array in combination with the Genome-Wide Human SNP Array 6.0, or alternatively the CytoScan HD Array (all from Affymetrix). The results were analyzed with the Chromosome Analysis Suite (Affymetrix), the breakpoint regions delimited by the array markers were examined in the UCSC Genome Browser (http://genome.ucsc.edu/; assembly NCBI37/hg19), and repetitive sequences in these regions were identified using RepeatMasker [24] within the Genome Browser. Breaks were pinpointed by sequencing as next described.

### Sequencing

PCR primers were designed using Primer3 software (http://frodo.wi.mit.edu/primer3/); primer sequences and PCR conditions are described in the Table S1. Sequencing was performed using BigDye Terminator v3.1 sequencing kits (Applied Biosystems, USA). Electrophoresis was carried out on an ABI 3730 xl DNA analyzer (Applied Biosystems, USA).

Statistical analyses

Association tests were performed using two-degrees of freedom Pearson's chi-square test with Yates correction. Statistical analyses were performed using the statistical package *stat*s with the software R v3.0.2. A nominal P-value of 0.05 was considered significant.

## Results

Among the 651 apparently unrelated index cases studied we found four different LGRs, three in *BRCA1* by MLPA and one in *BRCA2* by sequencing (Table 1).

**Table 1:** Large genomic rearrangements in the *BRCA1* gene identified in the Galician population.

Family	Gene	BIC LGR designation	Clinical Significance	Detection method	Confirmation method	Other affected genes	HGVS designation^a^	Size (bp)	Previously reported LGR affecting the same exons	Size (bp)	Geographical region
I	*BRCA1*	exon1-2dup	VUS	MLPA	CNV/SNP array, sequencing	*NRB2*	NG_005905.2: g.90012_97270dup	7,259	Del Valle et al. [12]	nd	Spain
II, III	*BRCA1*	exon1-24del	Deleterious	MLPA	CNV/SNP array, sequencing	*NRB2*	NG_005905.2: g.70536_180359del	109,824	De la Hoya [9]	nd	Spain
									Blay et al. [16]	nd	Asturias (Northern Spain)
									García-Casado et al. [25]	∼150,000	Spain
									Konecny et al. [26]	nd	Central Europe
									Engert et al. [27]	259,000- 345,000	Germany
IV	*BRCA1*	exon1-13del	Deleterious	MLPA	CNV/SNP array, sequencing	*NBR2, NBR1, TME106A*	NC_000017.10: g.41230935_41399840delinsAluSx1	168,905	Del Valle et al. [12]	∼250,000	Spain
									Blay et al. [16]	nd	Asturias (Northern Spain)
									Pylkas et al. [28]	nd	Finland
V	*BRCA2*	384insAlu	Deleterious	Sequencing	Primer-specific sequencing	–	NG_012772.1: g.8686_8687insAluYa5	∼350	Peixoto et al. [8]	∼350	Portugal

VUS: variant of uncertain significance; nd: non described.

a According to reference sequences NG_005905.2 or NG_012772.1, except for *BRCA1* exon1-13del, since it does not extend far enough upstream to describe the deletion of exons 1-13.

### Exon1-2dup

**Figure 1:**
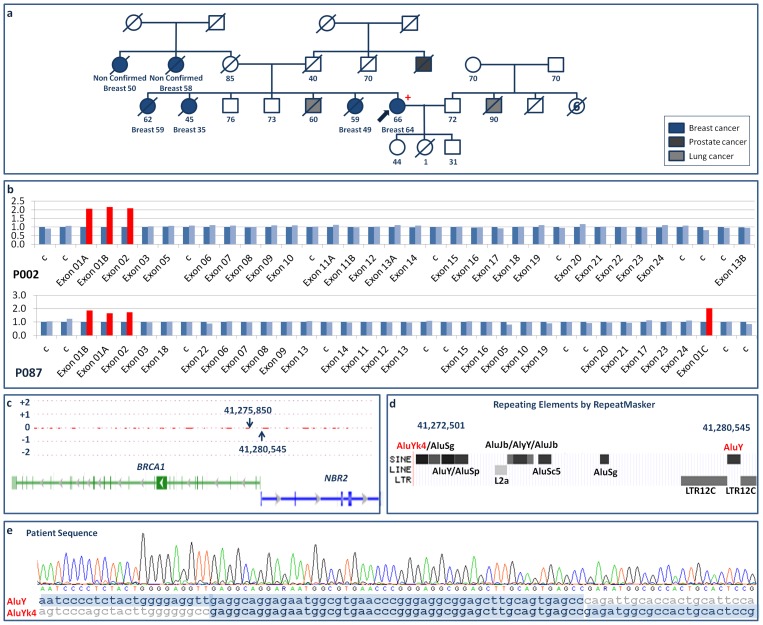
*BRCA1* exon1-2dup. a) Pedigree of Family I. +: mutation carrier; -: mutation non-carrier. b) MLPA normalized ratio results. Dark blue: reference signal for each probe created as described under Material and Methods. Light blue: sample probes with ratios ≥0.7 and ≤1.3. Red: sample probes with ratios >1.3 or <0.7. c) Location of the downstream breakpoint region on the forward strand, as delimited (arrows) by SNP array results. Genes on the forward strand are shown in blue and genes on the reverse strand in green. d) Repetitive elements identified by RepeatMasker in the breakpoint region. e) Overlapping AluY and AluYk4 sequences at the junction between the repeated sequences containing exons 1 and 2.

A duplication of *BRCA1* exons 1-2 was identified in a 66-year-old woman in whom breast cancer was diagnosed at age 64 years. Previously, her three sisters had developed breast cancer (at ages 35, 49 and 59 years), as had her two maternal aunts (according to the family, though this was not confirmed) (Family I, Fig. 1). The SNP array bracketed the downstream breakpoint but not the upstream one (Fig. 1c; throughout this paper, “upstream” and “downstream” respectively refer to the directions of decreasing and increasing genomic coordinates). However, sequencing identified a hybrid Alu element in which AluYk4 and AluY, two of the ten Alu elements identified by RepeatMasker in the bracketed region and its upstream vicinity (Fig. 1d), overlapped by 48 nt (Fig. 1e), indicating the tandem repetition of *BRCA1* exons 1A, 1B and 2 through duplication of the 7,259-nt sequence NG_005905.2:g.90012_97270. The origin of the duplication was thus an unequal homogolous recombination event that created the hybrid Alu element at the point of recombination. Note that since non-coding *BRCA1* exon 1A shares part of its sequence with non-coding *NBR2* exon 1 (the two exons together forming a bidirectional promoter regulated by different transcriptional repressor factors), the duplication of *BRCA1* exons 1 and 2 also means the duplication of part of the neighboring gene (*NBR2* exon 1). Unfortunately, a co-segregation study could not be performed since the affected family members were deceased.

### Exon1-24del

**Figure 2:**
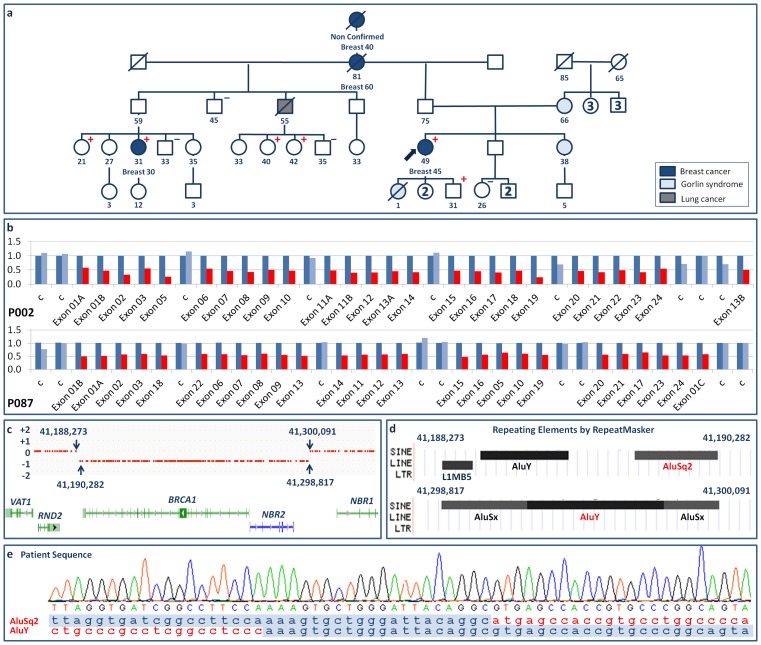
*BRCA1* exon1-24del. a) Pedigree of Family II. +: mutation carrier; -: mutation non-carrier. b) MLPA normalized ratio results (color key as for Fig. 1). c) Location of breakpoint regions on the forward strand, as delimited (arrows) by SNP array results (gene color key as for Fig. 1). d) Repetitive elements identified by RepeatMasker in the breakpoint regions. e) Overlapping AluY and AluSq2 sequences at the junction (red letters indicate deleted segments).

The complete deletion of *BRCA1* was identified in two presumably unrelated Galician families. In Family II the index patient was a 49-year-old woman in whom breast cancer was diagnosed at age 45 years (Fig. 2a). Breast cancer had also been diagnosed in her paternal grandmother (at age 60 years), in a half-cousin on her father's side (at age 30 years), and in her great-grandmother, although this last case was not confirmed. In Family III the deletion of exons 1-24 was detected in a 50-year-old woman who had sought genetic evaluation following identification of this deletion in her sister, in whom breast cancer had been diagnosed at age 46 years (the sister had been evaluated in another laboratory and pedigree data were not made available to us). In the present study, SNP array analysis showed that in both the affected families the deletion includes *NBR2* and *BRCA1* (Fig. 2c), and in both cases amplification and sequencing of the junction region identified a segment in which AluSq2 and AluY, two of the five Alu elements located by RepeatMasker in the breakpoint regions (Fig. 2d), shared a sequence of 20 nucleotides (Fig. 2e). We accordingly identify this LGR as the 109,824 bp deletion NG_005905.2:g.70536_180359del, and as attributable to unequal homologous recombination.

### Exon1-13del

**Figure 3:**
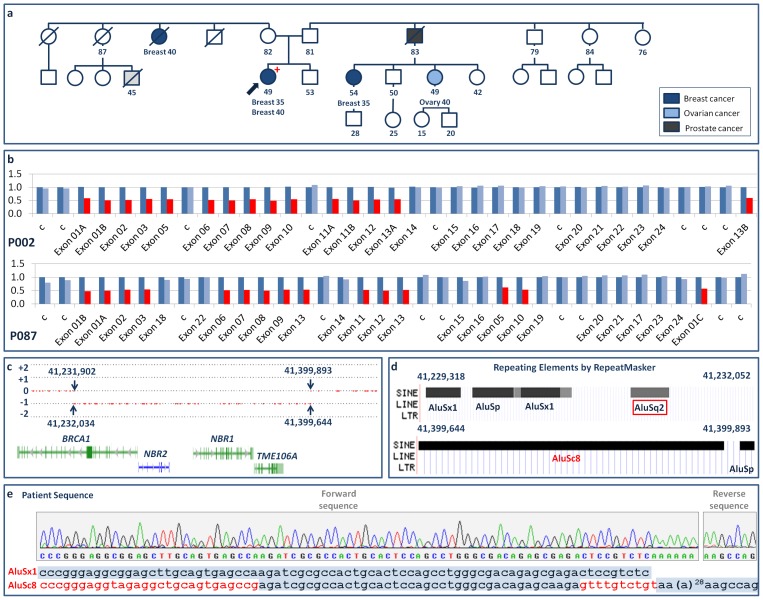
*BRCA1* exon1-13del. a) Pedigree of Family IV. +: mutation carrier; -: mutation non-carrier. b) MLPA normalized ratio results (color key as for Fig. 1). c) Location of breakpoint regions on the forward strand, as delimited (arrows) by SNP array results (gene color key as for Fig. 1). d) Repetitive elements identified by RepeatMasker in the breakpoint regions, with a red frame highlighting the AluSq2 element replaced by AluSx1 in the patient. e) Overlapping AluSx1 and AluSc8 sequences at the junction (red letters indicate deleted segments, and forward and reverse sequences are shown because of the poly-A tail of AluSc8).

Deletion of *BRCA1* exons 1-13 was detected in a 49-year-old woman in whom bilateral breast cancer was diagnosed at 35 and 40 years of age. One of her paternal cousins and a maternal aunt had also developed breast cancer (at ages 35 and 40 years, respectively), and another paternal cousin developed ovarian cancer at age 40 years (Fig. 3a). In the downstream breakpoint region delimited by the SNP array (Fig. 3c) RepeatMasker showed two Alu elements (Fig. 3d). In the putative upstream breakpoint region it found no repetitive elements, but did find several further upstream. Using a forward primer hybridizing on a non-repetitive sequence (Table S1), we were able to identify a segment in which one of the downstream Alu elements, AluSc8, shares 41 nucleotides with an AluSx1 (Fig. 3e), the initial 173-nt segment of which is homologous with the initial segment of AluSq2, the first upstream Alu element (see Figure S1). This LGR therefore seems to have arisen through an unequal homologous recombination event involving the deletion of the 168,905-bp sequence NC_000017.10:41230935_41399840 and its replacement with AluSx1 (doubtless favored by the homology with AluSq2).

### 384insAlu

**Figure 4:**
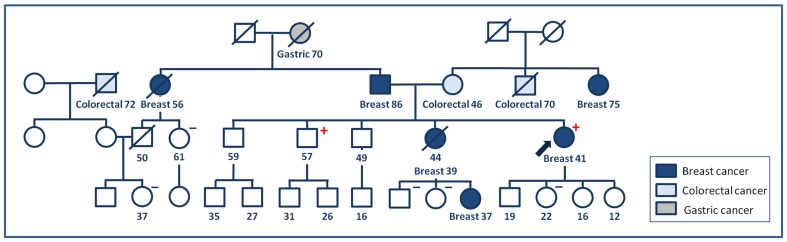
*BRCA2* c.156_157insAluYa5. Pedigree of Family V. +: mutation carrier; -: mutation non-carrier.

Routine screening detected the Portuguese founder mutation *BRCA2* c.156_157insAluYa5 in a Galician woman in whom breast cancer was diagnosed at 41 years of age. Her niece, her deceased sister, her father and two aunts (one on each side) had also developed breast cancer (Fig. 4).

In the various studies describing *BRCA1* and/or *BRCA2* LGRs in diverse regions of the Iberian Peninsula (Table 2), the reported frequency of *BRCA1* LGRs among HBOC families without point mutations or small indels ranges from 0.48% to 2.33%, and that of *BRCA2* LGRs from 0% to 8.4%. The difference between the deleterious LGRs frequency in Galicia and in Spanish population or in the rest Iberian populations is statistically significant (Table 3. Chi-square test with Yates correction *P*-value = 0.013 or 0.014, respectively).

**Table 2:** Frequency of *BRCA1* and *BRCA2* LGRs in the Iberian Peninsula.

					*BRCA1*					*BRCA2*							
						*N_LGR_*/*N_BRCA-_*		*N_LGR_*/*N_BRCA1+_*			*N_LGR_*/*N_BRCA-_*			*N_LGR_*/*N_BRCA2+_*			
Study	*N_BRCA-_*	*N_BRCA1+_*	*N_BRCA2+_*	Geographical region	*N_LGR_*	%	(95% CI)	%	(95% CI)	*N_LGR_*	%	(95% CI)	%		(95% CI)		
**De la Hoya et al. 2006 ** [9]	285	73	ne	Spain	6	2.11	(0.86-4.75)	8.22	(3.39–17.65)	ne							
**Gutierrez-Enríquez et al. 2007 ** [10] a	335	ne	na	Spain	ne					5	1.49	(0.55–3.65)	na				
**Miramar et al. 2008 ** [11]	44	8	0	Aragon (Northeast Spain)	1	2.27	(0.12–13.51)	12.50	(0.66–53.32)	0			0				
**Del Valle et al. 2010 ** [12] a	257	na	na	Spain	6	2.33	(0.95–5.26)	na		2	0.78	(0.14–3.09)	na				
**Rodriguez et al. 2010 ** [13]	207	na	na	Catalonia (Northeast Spain)	1	0.48	(0.03–3.01)	na		1	0.48	(0.03–3.08)	na				
**Peixoto et al. 2006 and 2011 ** [6], [8] b	79/131	15	19	Portugal	1	1.27	(0.07–7.82)	6.67	(0.35–33.97)	11	8.4	(4.47–14.87)	57.89		(33.97–78.88)		
**Ruiz de Garibay et al. 2012 ** [14]	813	ne	na	Spain	ne					7	0.86	(0.38–1.85)	na				
**Juan Jiménez et al. 2013 ** [15]	1471	155	155	Valencian Community (Eastern Spain)	17	1.16	(0.70–1.89)	10.97	(6.71–17.24)	1	0.07	(0.00–0.44)	0.65		(0.03–4.08)		
**Blay et al. 2013 ** [16]	200	36	0	Asturias (Northern Spain)	3	1.50	(0.39–4.68)	8.33	(2.18–23.59)	0			0				
**Present study**	651	128	55	Galicia (Northwest Spain)	4	0.61	(0.20–1.68)	2.34^c^	(0.61–7.22)	1	0.15	(0.01–0.99)	1.81		(0.10–11.18)		

*N_BRCA-_*: number of families without *BRCA1/2* point mutations or small indels included in the study. *N_BRCA1+_*: number of families with *BRCA1* mutations; *N_BRCA2+_*: number of families with *BRCA2* mutations; *N_LGR_*: number of families with LGRs; ne: non evaluated; na: non available.

a The number of families with point mutations or small indels was not stated for each gene.

b Data for *BRCA1* LGRs is extracted from Peixoto et al. 2006 [6], whereas data for *BRCA2* LGRs is extracted formPeixoto et al. 2011 [8].

c Excluding *BRCA1* exon1-2dup, considered as of unknown pathogenicity. If this LGR is pathogenic, this frequency becomes 3.10%.

**Table 3:** Frequency of *BRCA1* and *BRCA2* deleterious LGRs.

	Galicia			Spain^a^			Iberian population^b^			Galicia vs Spain	Galicia vs Iberian populations
	N	%	(95% CI)	N	%	(95% CI)	N	%	(95% CI)	*P*-value^c^	*P*-value^c^
**N_LGR_**	3	2.34	(0.61–7.22)	27	9.93	(6.76–14.27)	28	9.76	(6.69–13.94)	0.013	0.014
**N** ***_BRCA1_***_**+**_	128			272			287				

*N_LGR_*: number of families with LGRs; *N_F_BRCA1+_*: number of families with *BRCA1* mutations.

a Estimated from the reports performed in Spanish populations with available data [9], [11], [15], [16].

b Estimated from the reports performed in Iberian populations with available data [6], [7], [9], [11], [15], [16].

c Chi-square test with Yates correction *P*-value.

## Discussion

In the present study we have identified three LRGs in *BRCA1* and one in *BRCA2*. The duplication of exons 1 and 2, that is a variant of unknown significance, has been reported previously in a Spanish HBOC family, but was not characterized in detail [12]. Therefore, although it is plausible that both rearrangements share the same breakpoint, it cannot be assessed. The complete deletion of *BRCA1* has in the past been reported five times, three cases concerning Spaniards [9], [16], [25], one a Central European patient [26] and the fifth a German [27], but only in one of these cases, that identified a *de novo BRCA1* deletion, were the breakpoints characterized [25]. It is interesting that none of the three complete *BRCA1* deletions with published length estimates can share both breakpoints, their lengths being <110 kb (this work), 259–345 kb [27], and ∼150 kb [25] (this last deletion extending from the beginning of *NBR1* to *VAT1* and including the whole of *RND2*, *ψBRCA1*, *BRCA1* and *NBR2*). The deletion of *BRCA1* exons 1-13 has previously been reported in three families, two Spanish [12], [16] and one Finnish [28]. It is unclear whether either of the breakpoints of this deletion (NC_000017.10:41230935_41399840) coincides with or lies close to the corresponding breakpoint of the approximately 250 kb exon1-13del LGR reported by del Valle et al. [12]: both these deletions eradicate *NBR2*, *NBR1* and *TMEM106A* as well as *BRCA1* exons 1-13. However, the present LGR does not affect *ARL4D*, whereas Del Valle et al. [12] only identified their downstream breakpoint as lying somewhere between exon 6 of *TMEM106A* and exon 2 of *ARL4D*. Concerning the *BRCA2* c.156_157insAluYa5 mutation, Peixoto et al. [8] recently reported finding this mutation in only three out of 5,294 families living outside Portugal all three of which had emigrated relatively recently from Portugal. This is therefore, as far as we know, the first report of c.156_157insAluYa5 in a family not known to be of Portuguese origin. However, recent generations of our patient's family have resided near the frontier between Spain and northern Portugal, where a high frequency of this founder mutation has been reported [7]. Although the absence of Portuguese ancestors in the past four generations has been reported by the family, the estimated age of the mutation, 561±215 years (estimated by the study of 19 SNPs and nine microsatellite markers spanning ∼2 Mb within and around *BRCA2* [8]) makes plausible a Portuguese origin for the mutation in our family.

### Clinical classification of the identified LGRs

*BRCA1* deletions of exons 1-13 and 1-24 are considered pathological LGR [12]. In both cases the transcription start sites are removed, likely resulting in the lack of the transcript. Accordingly to Peixoto et al. [7], *BRCA2* c.156_157insAluYa5 is classified as deleterious since it results in exon 3 skipping and co-segregates with the disease. However, the pathogenicity of the duplication of *BRCA1* exons 1-2 cannot be assessed. Despite the efforts carried out by del Valle et al. [12] and by us to study the effect at the RNA level we were unable to amplify the aberrant allele. Moreover, a co-segregation study could not be performed in none of the families identified in each report. Therefore, this variant must remain as of uncertain significance.

### Homologous vs. non-homologous recombination as the origin of *BRCA1/2* LGRs

Having identified non-homologous recombination events as the sources of three of the four *BRCA2* LGRs they analyzed, Ruiz de Garibay et al. suggested that the proportion of *BRCA2* LGRs originated by homologous recombination had been overestimated [14]. For *BRCA1* LGRs, the present results are in keeping with statistics showing the predominant mechanism to be Alu-mediated homologous recombination [4]. Furthermore, all the Alu elements apparently involved in producing the *BRCA1* LGRs observed in the present study are members of the evolutionarily youngest subfamilies, AluS and AluY, which have a high degree of mutual homology [29].

### *BRCA1/2* LGRs in the Iberian Peninsula

Founder mutations are responsible for the high rates of LGRs in the Valencian Community (Eastern Spain), where NG_005905:g.97346_111983del has deleted *BRCA1* exons 3-5 in 10.97% of all families with mutations in the *BRCA1* gene [30], and in Portugal, where *BRCA2* c.156_157insAluYa5 (NG_012772:g.8686_8687ins AluYa5) accounts for 57.89% of all mutant *BRCA2* families [8]. By contrast, in our population deleterious LGRs constitute only 2.34% (95% CI: 0.61–7.22) of all families with definitely pathogenic *BRCA1* variants, given the small duplication exon1-2dup cannot be classified as a deleterious mutation. The observed frequency it is the lowest rate reported to date for *BRCA1* LGRs in an Iberian population. We should however note that given that most of the studies performed to date in Iberian populations are characterized by their limited sample size, the accuracy of the estimates is limited, as it is demonstrated by the wide interval of the frequency at 95% confidence level. Nonetheless, the frequency of *BRCA1* LGRs within *BRCA1* mutation carriers in Galician population seems to differ from Spanish (*P*-value = 0.013) and Iberian populations (*P*-value = 0.014).

### Role of MLPA in testing for *BRCA1/2* LGRs

MLPA is a fast, sensitive means of detecting LGRs, and cannot at present be replaced by massively parallel sequencing methods: recent studies suggest that these latter are adequate for detection of point mutations of *BRCA1* and *BRCA2*, but are insufficiently specific for LGRs [31]. However, the optimization of next generation sequencing standard protocols for detection of Alu element rearrangements have resulted in false positive reads [32]. The shortcoming of MLPA is that it does not identify LGR breakpoints, as is necessary for recognition of recurrent rearrangements, for inference of the molecular mechanisms of rearrangement, and for rapid analysis of a proband's relatives. Breakpoint identification still requires other strategies, such as methods based on Sanger sequencing. Another question is whether MLPA should be performed before or after screening for point mutations and small indels. Given the relatively high frequency of LGRs they found among *BRCA1* mutations in Spain, 8.2%, de la Hoya et al. [9] suggested that screening for LGRs by MLPA should be the first test performed in the evaluation of *BRCA1* in Spanish subjects, since this would speed results for a considerable number of families. Others have proposed the first-line use of MLPA on the grounds of cost effectiveness [33], [34]. However, the low frequency of LGRs found in the present study among Galician families with *BRCA1/2* mutations does not justify this strategy in this region.

In conclusion, we have detected three *BRCA1* large rearrangements in four families and have determined their breakpoints. Two of these three LGRs, which are classified as deleterious mutations, account for 0.61% of referrals without point mutations or small indels in *BRCA1*, and 2.34% of all families with *BRCA1* mutations in Galicia, the lowest figure reported to date in the Iberian population. All three involve Alu elements, which corroborates the predominance of Alu-mediated mechanisms in the production of *BRCA1* LGRs. LGRs affecting the same exons have been reported previously, but without breakpoint determination, and in some cases cannot have coincided with those observed in this study. We also detected one *BRCA2* LGR, the Portuguese founder mutation c.156_157insAluYa5. To our knowledge, this is the first time this mutation has been detected in a family not known to be of Portuguese origin. However, a distant Portuguese ancestry cannot be ruled out.

## Supporting Information

Figure S1 **AluSq2 replacement by AluSx1 in NC_000017.10:g.41230935_41399840delinsAluSx1.** a) Patient's electropherogram. b) Reference sequence, patient sequence, and AluSx1 sequence (Repbase Sequences). c) Blastn suite.
(DOCX)

Table S1 **Primer sequences and PCR conditions.**
(DOCX)
