# Supplementary material for: Large Genomic Rearrangements of BRCA1 and BRCA2 among Patients Referred for Genetic Analysis in Galicia (NW Spain): Delimitation and Mechanism of Three Novel BRCA1 Rearrangements
Source: PLoS One. 2014 Mar 31;9(3):e93306. doi: 10.1371/journal.pone.0093306 (PMC3970959; doi:10.1371/journal.pone.0093306)
Supplement: Figure S1 — AluSq2 replacement by AluSx1 in NC_000017.10:g.41230935_41399840delinsAluSx1. a) Patient's electropherogram. b) Reference sequence, patient sequence, and AluSx1 sequence (Repbase Sequences). c) Blastn suite. (DOCX) [file pone.0093306.s001.docx]

**Figure S1.** AluSq2 replacement by AluSx1 in NC_000017.10:g.41230935_41399840delinsAluSx1

**a) Patient´s electropherogram**


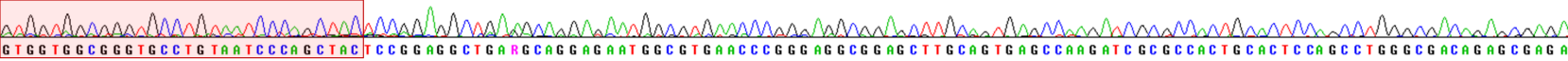


Red box highlights the homologous region between AluSq2 and AluSx1

**b) Reference sequence, patient sequence, and AluSx1 sequence (Repbase Sequences)**

Capital letters: non repetitive sequence

Minor letters: repetitive element

Red minor letters highlights the homologous region between AluSq2 and AluSx1

>Reference Sequence (NC_000017.10)

CCCCCAATACAGGGTGATAATTGATAAAGGGTAATGTGCAAGTTCCAAGGAACCATATCAAACGGAATTAACCATTGGAAAGAGTTAAAAAAGTggccaggtgatgtggctcacgcctacaatcccagcactttgagaggccgaggtggacagatcacctgaggtcaggagtttgagaccagcccggccaacatggtgaaaccctgtctctactaaaaatacaaaaattagctgggcgtggtggcgggtgcctgtaatcccagctacccaggaggctgaggcggacattgtaatgagccgaaatcacaccattgcactccagcctgggcaacaaaagtgaaacttcatctcaaacaaaaacaaaacaaaacaaaacaaaaaaaa

>Patient Sequence

CCCCCAATACAGGGTGATAATTGATAAAGGGTAATGTGCAAGTTCCAAGGAACCATATCAAACGGAATTAACCATTGGAAAGAGTTAAAAAAGTggccaggtgatgtggctcacgcctacaatcccagcactttgagaggccgaggtggacagatcacctgaggtcaggagtttgagaccagcccggccaacatggtgaaaccctgtctctactaaaaatacaaaaattagctgggcgtggtggcgggtgcctgtaatcccagctactccggaggctgagagcaggagaatggcgtgaacccgggaggcggagcttgcagtgagccaagatcgcgccactgcactccagcctgggcgacagagcgagactccgtctca

>AluSx1, repbase; DNA; PRI; 283 BP; SINE1/7SL SINE from primates.SINE1/7SL; SINE; Non-LTR Retrotransposon; Transposable Element; AluSx1

ggccgggcgcggtggctcacgcctgtaatcccagcactttgggaggccgaggcgggcggatcacctgaggtcaggagttcgagaccagcctggccaacatggtgaaaccccgtctctactaaaaatacaaaaattagccgggcgtggtggcgggcgcctgtaatcccagctactcgggaggctgaggcaggagaatcgcttgaacccgggaggcggaggttgcagtgagccgagatcgcgccactgcactccagcctgggcgacagagcgagactccgtctca

**c) Blastn suite**

Ref.Seq 1 CCCCCAATACAGGGTGATAATTGATAAAGGGTAATGTGCAAGTTCCAAGGAACCATATCA 60

Pat.seq 1 CCCCCAATACAGGGTGATAATTGATAAAGGGTAATGTGCAAGTTCCAAGGAACCATATCA 60

Ref.Seq 61 AACGGAATTAACCATTGGAAAGAGTTAAAAAAGTGGCCAGGTGATGTGGCTCACGCCTAC 120

Pat.seq 61 AACGGAATTAACCATTGGAAAGAGTTAAAAAAGTGGCCAGGTGATGTGGCTCACGCCTAC 120

AluSx1.Ref 1 GGCCGGGCGCGGTGGCTCACGCCTGT 26

Ref.Seq 121 AATCCCAGCACTTTGAGAGGCCGAGGTGGACAGATCACCTGAGGTCAGGAGTTTGAGACC 180

Pat.seq 121 AATCCCAGCACTTTGAGAGGCCGAGGTGGACAGATCACCTGAGGTCAGGAGTTTGAGACC 180

AluSx1.Ref 27 AATCCCAGCACTTTGGGAGGCCGAGGCGGGCGGATCACCTGAGGTCAGGAGTTCGAGACC 86

Ref.Seq 181 AGCCCGGCCAACATGGTGAAACCCTGTCTCTACTAAAAATACAAAAATTAGCTGGGCGTG 240

Pat.seq 181 AGCCCGGCCAACATGGTGAAACCCTGTCTCTACTAAAAATACAAAAATTAGCTGGGCGTG 240

AluSx1.Ref 87 AGCCTGGCCAACATGGTGAAACCCCGTCTCTACTAAAAATACAAAAATTAGCCGGGCGTG 146

Ref.Seq 241 GTGGCGGGTGCCTGTAATCCCAGCTACCCAGGAGGCT----------------------- 277

Pat.seq 241 GTGGCGGGTGCCTGTAATCCCAGCTACTCCGGAGGCTGAGAGCAGGAGAATGGCGTGAAC 300

AluSx1.Ref 147 GTGGCGGGCGCCTGTAATCCCAGCTACTCGGGAGGCTGAGGCAGGAGAATCGCTTGAACC 206

Ref.Seq 278 ----GAGGCGGACATTGTAATGAGCCGAAATCACACCATTGCACTCCAGCCTGGGCAACA 333

Pat.seq 301 CCGGGAGGCGGAGCTTGCAGTGAGCCAAGATCGCGCCACTGCACTCCAGCCTGGGCGAC- 359

AluSx1.Ref 207 CGG-GAGGCGGAGGTTGCAGTGAGCCGAGATCGCGCCACTGCACTCCAGCCTGGGCGAC- 264

Ref.Seq 334 AAAGTGAAACTTCATCTca 352

Pat.seq 360 AGAGCGAGACTCCGTCTCA 378

AluSx1.Ref 265 AGAGCGAGACTCCGTCTCA 283
