# Supplementary material for: Large Genomic Rearrangements of BRCA1 and BRCA2 among Patients Referred for Genetic Analysis in Galicia (NW Spain): Delimitation and Mechanism of Three Novel BRCA1 Rearrangements
Source: PLoS One. 2014 Mar 31;9(3):e93306. doi: 10.1371/journal.pone.0093306 (PMC3970959; doi:10.1371/journal.pone.0093306)
Supplement: Table S1 — Primer sequences and PCR conditions. (DOCX) [file pone.0093306.s002.docx]

**Table S1:** Primer sequences and PCR conditions

| **BIC LGR designation** | **PCR Primer** | **Sequence** | **Annealing Temperature** | **Sequencing Primer** | **Sequence** |
| --- | --- | --- | --- | --- | --- |
| **exon1-24del ^a^** | BRCA1del1-24F | CCATTGTCGTTCATTTCACTCC | 64 | BRCA1del1-24F | CCATTGTCGTTCATTTCACTCC |
|  | BRCA1del1-24R | TTCCTCTAAAGTGGCAGGAAACA |  | BRCA1del1-24R | TTCCTCTAAAGTGGCAGGAAACA |
| **exon1-2dup ^a^** | BRCA1dup1-2F | CAACAGGATGTGGGTGGGGT | 64 | BRCA1dup1-2F | CAACAGGATGTGGGTGGGGT |
|  | BRCA1dup1-2R | GTGGTAGGATCACGAGGTCAGG |  | BRCA1dup1-2R | GTGGTAGGATCACGAGGTCAGG |
| **exon1-13del ^b^** | BRCA1del1-13F4 | GAAAGAGTACCGGAGAGCACAAA | 64 | BRCA1del1-13F8 | CCCCCAATACAGGGTGATAA |
|  | BRCA1del1-13R3 | AGGGGTGGACCCTACATTATCTC |  | BRCA1del1-13R3 | AGGGGTGGACCCTACATTATCTC |

^a^ PCR was performed using GoTaq DNA Polymerase (Promega) according to manufacturer´s protocol.

^b^ PCR was performed using Expand Long Template PCR System (Roche) according to manufacturer´s protocol.
